# Early life blood lead levels and asthma diagnosis at age 4–6 years

**DOI:** 10.1186/s12199-021-01033-0

**Published:** 2021-11-12

**Authors:** Marina Oktapodas Feiler, Carly J. Pavia, Sean M. Frey, Patrick J. Parsons, Kelly Thevenet-Morrison, Richard L. Canfield, Todd A. Jusko

**Affiliations:** 1 Department of Epidemiology and Biostatistics, College of Public Health, Temple University, 1301 Cecil B. Moore Ave, Philadelphia, PA 19122 USA; 2 Department of Environmental Medicine, University of Rochester Medical Center, University of Rochester, 601 Elmwood Ave, Rochester, NY 14642 USA; 3 Ramboll US Consulting Inc., 201 California St. #1200, San Francisco, CA 94111 USA; 4 Department of Pediatrics, University of Rochester Medical Center, University of Rochester, 265 Crittenden Blvd, Rochester, NY 14642 USA; 5 Division of Environmental Health Sciences, Wadsworth Center, New York State Department of Health, Empire State Plaza, Albany, NY 12201 USA; 6 Department of Environmental Health Sciences, State University of New York at Albany, 1 University Pl, Rensselaer, NY 12144 USA; 7 Department of Public Health Sciences, University of Rochester Medical Center, University of Rochester, 265 Crittenden Blvd, Rochester, NY 14642 USA; 8 Division of Nutritional Sciences, Cornell University, M Van Rensselaer Hall, Ithaca, NY 14853 USA

**Keywords:** Metals, Respiratory disease, Pediatric, Rochester

## Abstract

The USA has a high burden of childhood asthma. Previous studies have observed associations between higher blood lead levels and greater hypersensitivity in children. The objective of the present study was to estimate the association between blood lead concentrations during early childhood and an asthma diagnosis between 48 and 72 months of age amongst a cohort with well-characterized blood lead concentrations. Blood lead concentrations were measured at 6, 12, 18, 24, 36, and 48 months of age in 222 children. The presence of an asthma diagnosis between 48 and 72 months was assessed using a questionnaire which asked parents or guardians whether they had been told by a physician, in the past 12 months, that their child had asthma. Crude and adjusted risk ratios (RR) of an asthma diagnosis were estimated for several parameterizations of blood lead exposure including lifetime average (6 to 48 months) and infancy average (6 to 24 months) concentrations. After adjustment for child sex, birthweight, daycare attendance, maternal race, education, parity, breastfeeding, income, and household smoking, age-specific or composite measures of blood lead were not associated with asthma diagnosis by 72 months of age in this cohort.

## Introduction

An estimated 6.2 million children < 18 years old have asthma in the USA [1]. Asthma is a complex disease caused by genetic and environmental factors [2]. The most prevalent form of childhood asthma, IgE-mediated allergic disease, is caused by an imbalance of T-helper (T_H_) cells favoring the production of T_H_2 and associated cytokines [3]. Environmental toxicants that interfere with T cell balance may play a role in the pathogenesis of asthma. For example, air pollution and environmental tobacco smoke (ETS), known risk factors of asthma [4, 5], can decrease the function of regulatory T cells and contribute to greater asthma-related cytokine production [6, 7].

Experimental results [8–11] demonstrate that lead toxicity causes an imbalance in T_H_ function, in favor of the T_H_2 response and IgE production, consistent with hypersensitive immune dysfunction. However, epidemiological studies that directly evaluate the risk of lead on asthma and other hypersensitivity phenotypes have been limited by cross-sectional designs, single measures of lead exposure, or potential residual confounding [12–19]. The objective of this study is to address these gaps by estimating the association between environmental lead exposure at multiple time points during early childhood and asthma diagnosis by 72 months of age in a well-characterized urban cohort.

## Methods

### Study sample

The study sample of 222 children is a subset of a cohort of 276 children born between July 1994 and January 1995 who were enrolled in a dust control intervention trial at 6 months of age [20]. In order to be enrolled in the trial, children (5–7 months old at baseline) had to reside in Rochester, New York and families must have had no plans to relocate for at least 3 months [20]. The 222 children included in this sub-analysis had complete information on asthma diagnosis and other covariables of interest. Parents/guardians were asked whether their child had been diagnosed with asthma by a physician in the past 12 months; children with a response at either 48, 60, or 72 months of age to this question were included in this study sample. Parents or guardians provided written consent and the study protocol was approved by the Institutional Review Board at the University of Rochester Medical Center, Rochester, New York.

### Asthma diagnosis

The outcome of interest in this study was parental/guardian report of a physician diagnosis of asthma in the preceding 12 months. This information was collected from a self-report questionnaire at the child’s 48-, 60-, and 72-month follow-up visits that asked, “In the past 12 months did the doctor say he/she [the child] had asthma?” Children were considered to have physician-diagnosed asthma if their parent/guardian reported a diagnosis at any one of these three time points.

### Blood lead measurement

Venous blood samples were collected when children were 6, 12, 18, 24, 36, 48, 60, and 72 months of age [20, 21]. All blood samples were analyzed for lead by electrothermal atomic absorption spectrometry (ETAAS) at the New York State Department of Health (NYSDOH) Wadsworth Center’s Trace Elements laboratory, a reference laboratory for blood lead. Blood lead was measured using a Perkin Elmer Model 400ZL atomic absorption spectrometry equipped with a transversely heated graphite atomizer (THGA) and longitudinal Zeeman background correction (PerkinElmer Life and Analytical Sciences, Shelton, CT). The THGA instrument was calibrated daily before each run with aqueous lead standards traceable to the National Institute of Standards and Technology (NIST, Gaithersburg, MD). Blood samples and QC materials were diluted 1 + 9 with reagents and 12 μL samples injected into the atomizer. Further details on this method, validation, and re-validation have been previously published [22, 23]. The limit of detection was estimated at ~ 1.0 μg/dL, with a limit of quantification at ~3.0 μg/dL. The standard deviation of repeatability of measurements ranged from 0.1 to 0.3 μg/dL for blood lead concentrations less than 10 μg/dL, and varied by less than 2% for blood lead measurements above 20 μg/dL [23]. Three concentrations of NYSDOH (Albany, NY) blood-based reference materials (including one < 10 μg/dL) were analyzed before, during, and after each analytical run as part of the laboratory’s internal quality assurance program [23].

Average and peak blood lead concentrations were calculated from serial blood lead measures, similar to previous analyses in this cohort [21, 24]. Lifetime average blood lead level was computed by dividing the total area under each child’s age-by-blood-lead curve by 42 (42 = 48–6 months), and infancy average blood lead concentration was computed by dividing the total area under the child’s age-by-blood-lead curve by 18 (18 = 24–6 months). Peak infancy concentration is the child’s highest blood lead level measured from 6 to 24 months and peak 48-month concentration is the child’s highest blood lead level measured from 6 to 48 months. Infancy blood lead measures were included in the analysis due to the rapid development of the immune system during this timeframe and, thus, may serve as a critical window for exposure to lead [8, 25]. Concurrent blood lead level is the measurement taken on the day of the 48-month visit. As in previous analyses, missing age-specific blood lead levels were imputed using conditional means regression, utilizing the values of non-missing blood lead levels [21]. The percentage of missing and imputed blood lead values were 2%, 7%, 8%, 14%, 16%, 19%, 14% and 4% at 6, 12, 18, 24, 36, 48, 60, and 72 months, respectively. In general, blood lead concentrations were moderately to strongly correlated, ranging from *r* = 0.21 (*p* < 0.05) between 6 and 24 months and *r* = 0.89 (*p* < 0.05) at 60 and 72 months, supporting the use of imputation.

### Data collection

At each visit, parents/guardians completed a questionnaire concerning the child’s health history, parental health, socioeconomic status, demographic information, and the home environment. Covariates of interest included data collected from the child’s birth record such as sex, birthweight (grams), and maternal parity. Maternal race (white/non-white) and whether the child was ever breastfed were collected at the child’s 6-month visit. The average adjusted income variable used in the analysis incorporated household income as well other sources of income (e.g., child support or government assistance) reported at the 54- through 72-month visits. This variable was calculated by averaging household income reported at 54, 60, 66, and 72 months, adding any average other income reported from that same time period, and subtracting out the average rent paid during that same period. Data collected at the child’s 72-month follow-up included maternal education (less than high school, high school/general education development (GED), more than high school), total number of cigarettes smoked per day in the household, and any child daycare attendance in the past 12 months.

### Statistical analyses

Statistical models included blood lead concentration as a continuous variable or as a three-level ordinal classification variable with categories ≤ 5.0 μg/dL, 5.0–9.9 μg/dL, and ≥ 10.0 μg/dL. These groupings were chosen based on recent Centers for Disease Control and Prevention (CDC) reference levels, clinical utility, and as having significance for informing public health policy [26, 27]. For instance, a goal of Healthy People 2020 was to eliminate blood lead levels greater than 10 μg/dL, and presently, the CDC considers 5 μg/dL as an action level for the introduction of monitoring of the child and remediation in the home [27]. Candidate confounders were identified from the existing literature [14, 17, 18, 28–33]. To identify the minimally sufficient set of adjustment variables, a directed acyclic graph (DAG) was constructed using DAGitty software (version 3.0) [34]. The minimally sufficient set of variables identified from the DAG were child sex, birthweight, daycare attendance, maternal race, education, parity, ever having breastfed, average adjusted income, and total number of cigarettes smoked per day in the household.

The risk ratio for asthma and respective 95% confidence interval (CI) were estimated using log-binomial regression for each parameterization of lead. In total, 10 primary models were fit: five unadjusted, and five adjusted, examining (1) concurrent 48-month blood lead levels, (2) peak 48-month blood lead levels, (3) lifetime average at 48-month blood lead levels, (4) peak 24-month blood lead levels, and (5) infancy average at 24-month blood lead levels. As a secondary analysis, continuous parametrizations of age-specific lead measurements at 6, 12, 18, 24, 36, 48, 60, and 72 months of age were examined. Risk ratios and *p* values for linear trend were considered statistically significant if *p* < 0.05. All data management and analyses were performed using the SAS software system (SAS Institute Inc., Cary, NY, USA; version 9.4).

## Results

**Fig. 1:**
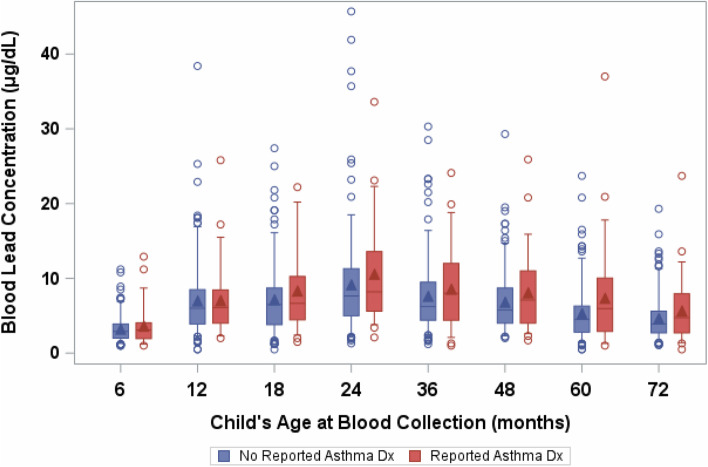
Distributions of blood lead concentrations for children with and without a reported asthma diagnosis (Dx) through 72 months of age. The outer limits of the boxes (top to bottom) represent the 75th and 25th percentiles; the horizontal bars within the boxes represent the 50th percentiles, and the triangles represent the means. The whiskers indicate 90^th^ and 10^th^ percentiles; observations outside of the 90^th^ and 10^th^ percentiles are represented as points

Among the 222 children with complete data, 70 (31%) had a concurrent blood level at 48 months < 5 μg/dL, 99 (45%) had a level between 5 and 9.9 μg/dL, and 53 (24%) had a level ≥ 10 μg/dL (Table 1). Nearly 45% of children had an infancy average lead level between 5 and 9.9 μg/dL, with 18% having a value ≥ 10 μg/dL. By 24 months of age, 92 (41%) of children had a peak value ≥ 10 μg/dL. Median lead concentrations ranged from 3 to 8 μg/dL, and, on average, blood lead concentrations increased through age 24 months, and decreased thereafter (Fig. 1).

**Table 1:** The distribution of various child, maternal, socio-economic, and lifestyle characteristics of interest in the total study sample and by lifetime average blood lead levels from 6 to 48 months of age, and asthma diagnosis (*n* = 222)

**Characteristics**	**Study sample** ***n*** **= 222** ***n*** **(%)**	**Lifetime average blood lead levels** **(μg/dL)**			**Asthma** **diagnosis** ***n*** **= 52** ***n*** **(%)**
		**< 5** ***n*** **= 70** ***n*** **(%)**	**5–9.9** ***n*** **= 99** ***n*** **(%)**	**≥ 10** ***n*** **= 53** ***n*** **(%)**	
**Child sex**					
Male	113 (51)	30 (43)	47 (47)	36 (68)	34 (65)
Female	109 (49)	40 (57)	52 (53)	17 (32)	18 (35)
**Birthweight (grams)**					
< 2500	15 (7)	6 (9)	7 (7)	2 (4)	3 (6)
2500–4000 (normal)	195 (88)	57 (81)	89 (90)	49 (92)	46 (88)
≥ 4000	12 (5)	7 (10)	3 (3)	2 (4)	3 (6)
Median	3270	3326	3203	3270	3326
(IQR)	(2891–3570)	(2850–3620)	(2850–3620)	(2976–3570)	(2990–3662)
**Maternal race**					
White	59 (27)	33 (47)	21 (21)	5 (9)	12 (23)
Non-white	163 (73)	37 (53)	78 (79)	48 (91)	40 (77)
**Maternal age at birth (years)**					
<20	68 (31)	19 (27)	33 (33)	16 (30)	13 (25)
20-24	45 (20)	12 (17)	21 (21)	12 (23)	14 (27)
25–30	61 (27)	21 (30)	23 (23)	17 (32)	16 (31)
≥ 30	48 (22)	18 (26)	22 (22)	8 (15)	9 (17)
Median (IQR)	24.6 (19.1–29.3)	26.1 (19.6–30.7)	23.6 (19.3–29.8)	23.9 (18.4–27.5)	24.2 (20.2–29.0)
**Maternal education**					
Less than high school	61 (27)	12 (17)	28 (28)	21 (40)	16 (31)
High school/GED	77 (35)	24 (34)	33 (33)	20 (38)	20 (38)
More than high school	84 (38)	34 (49)	38 (38)	12 (22)	16 (31)
**Maternal parity at 72 months of age**					
0	79 (36)	33 (47)	33 (33)	13 (25)	18 (35)
1	56 (25)	17 (24)	25 (25)	14 (26)	13 (25)
2	40 (18)	10 (14)	20 (20)	10 (19)	6 (12)
3	30 (14)	9 (13)	13 (13)	8 (15)	8(15)
≥ 4	17 (8)	1 (2)	8 (8)	8 (15)	7 (13)
**Annual household income at 72 months of age**					
< 24,999	155 (70)	34 (49)	74 (76)	47 (90)	41 (79)
≥ 25,000	65 (30)	36 (51)	24 (24)	5 (10)	11 (21)
**Household smoking exposure at 72 months of age**					
No	116 (52)	45 (64)	45 (45)	26 (49)	25 (48)
Yes	106 (48)	25 (36)	54 (55)	27 (51)	27 (52)
**Child ever breastfed by 6 months of age**					
No	184 (83)	55 (79)	82 (83)	47 (89)	49 (94)
Yes	38 (17)	15 (21)	17 (17)	6 (11)	3 (6)
**Daycare attendance at 72 months of age**					
No	140 (63)	47 (67)	57 (58)	36 (68)	34 (65)
Yes	82 (37)	23 (33)	42 (42)	17 (32)	18 (35)

Fifty-two children (23%) had been told by a doctor that they had asthma in the last 12 months during at least one of the three visits at 48, 60, or 72 months (Table 1). Children with higher blood lead levels at the 48-month visit were more likely to be male and reside in households with lower annual incomes and smoke exposure (Table 1). Mothers of children with higher blood lead levels tended to have more previous pregnancies, self-report non-white race, have less education, and report lower household income, compared with mothers of children with lower blood lead levels.

Table 2 reports the risk ratio between blood lead exposures and self-reported asthma diagnosis by a physician. Consistent with Fig. 1, unadjusted estimates were somewhat suggestive of an increased risk of asthma diagnosis among children with higher blood lead levels; however, results were not statistically significant (Table 2). After adjustment for potential confounders and risk factors, risk ratios were attenuated towards the null. For instance, in the crude model, children with a 48-month blood lead level ≥ 10 μg/dL had 1.56 times the risk of an asthma diagnosis (95% CI 0.89, 2.75) compared with children with a blood lead level < 5 μg/dL (Table 2). After adjusting for potential confounders, the association attenuated (RR = 1.14, 95% CI 0.60, 2.15) (Table 2). Overall, there was not strong evidence of a linear dose—response in categorical blood lead models, consistent with non-statistically significant p-values for linear trend. Adjusted risk ratios for age-specific 6-, 12-, 18-, 24-, 36-, 60-, and 72-month blood lead measurements did not show an association between lead and asthma, and all estimated risk ratios for each 1 μg/dL difference in blood lead were between 0.97 and 1.07 (data not shown in tabular form).

**Table 2:** Risk ratios (RRs) between blood lead levels and reported asthma diagnosis by a physician (*n* = 222)

Lead (μg/dL)	Asthma (***n*** = 52) ***n*** (%)	No asthma (***n*** = 170) ***n*** (%)	RR (95% CI)	aRR^**a**^ (95% CI)
**Concurrent at 48 months**				
< 5	19 (37)	64 (38)	1.00	1.00
5–9.9	18 (35)	79 (47)	0.81 (0.46, 1.44)	0.66 (0.36, 1.21)
≥ 10	15 (29)	27 (16)	1.56 (0.89, 2.75)	1.14 (0.60, 2.15)
*P* for linear trend ^b^			0.04	0.25
Continuous^c^			1.04 (1.00, 1.08)	1.03 (0.98, 1.09)
**Peak at 48 months**				
< 5	4 (8)	20 (12)	1.00	1.00
5–9.9	22 (42)	78 (46)	1.32 (0.50, 3.47)	1.34 (0.44, 4.09)
≥ 10	26 (50)	72 (42)	1.59 (0.61, 4.13)	1.31 (0.43, 3.98)
*P* for linear trend			0.21	0.58
Continuous			1.02 (0.99, 1.04)	1.01 (0.98, 1.04)
**Lifetime average at 48 months**				
< 5	15 (29)	55 (32)	1.00	1.00
5–9.9	18 (35)	81 (48)	0.85 (0.46, 1.57)	0.81 (0.43, 1.55)
≥ 10	19 (37)	34 (20)	1.67 (0.94, 2.97)	1.30 (0.68, 2.48)
*P* for linear trend			0.15	0.52
Continuous			1.03 (0.99, 1.08)	1.02 (0.96, 1.08)
**Peak at 24 months**				
< 5	4 (8)	28 (17)	1.00	1.00
5–9.9	23 (44)	75 (44)	1.88 (0.70, 5.02)	2.11 (0.69, 6.42)
≥ 10	25 (48)	67 (39)	2.17 (0.82, 5.77)	2.03 (0.67, 6.16)
*P* for linear trend			0.16	0.46
Continuous			1.02 (0.99, 1.04)	1.01 (0.98, 1.05)
**Infancy average at 24 months**				
< 5	18 (35)	66 (39)	1.00	1.00
5–9.9	19 (36)	80 (47)	0.90 (0.50, 1.59)	0.89 (0.50, 1.58)
≥ 10	15 (29)	24 (14)	1.79 (1.01, 3.17)	1.42 (0.76, 2.66)
*P* for linear trend			0.25	0.73
Continuous			1.03 (0.98, 1.08)	1.01 (0.95, 1.07)

^a^Adjusted for child sex, child birthweight, maternal race, maternal parity, maternal education, number of cigarettes smoked in the home per day at 72 months visit, average adjusted household income from 56 to 72 months visit, day care attendance at 72 months visit, and any breastfeeding by 6 months of age

^b^Tests of linear trend for categorical blood lead variables were based on the *p* value for the continuous, quantitative parameterizations of each blood lead variable

^c^Risk ratios for continuous models represent the risk of asthma diagnosis for every 1 μg/dL increase in blood lead concentrations

## Discussion

The objective of the present study was to determine whether blood lead levels in early childhood are associated with caregiver reported asthma by 72 months. In this well-characterized cohort of children with multiple blood lead measures taken during infancy and childhood and repeated assessments of asthma diagnosis, we did not observe evidence of an association between caregiver-reported asthma and any of the measures of childhood lead exposure, including infancy average lead exposure or age-specific measures of lead taken during infancy.

In this cohort, median lead concentrations ranged from 3 to 8 μg/dL, and blood lead concentrations tended to increase from 6 to 24 months of age and decreased thereafter. Blood lead levels in this study sample are elevated compared with recent national averages reported from the National Health and Nutritional Examination Survey (NHANES) [35]. Our study has higher blood levels for two main reasons: (1) compared with the general population, the urban residents of Rochester, NY are exposed to higher levels of lead from a high burden of lead paint in older housing and rental properties across the City, and (2) the present study has measured lead values when, on average, blood lead levels were higher in the U.S. population, and have steadily declined over time [26]. The increase and then decrease in median blood lead levels we observed over time in this cohort are expected given that children engage in increasing hand-to-mouth behaviors from age 6 months throughout the second year of life, increasing their exposure to dust, soil, and other sources of lead [36].

Previous epidemiological studies have observed higher IgE concentrations among children with higher blood lead levels, with mean blood lead levels in the range of 1–2 μg/dL [13, 14, 33]. A study of 279 children aged 9 months to 6 years in the USA observed a positive correlation (*r* = 0.22, *p* = 0.0004) between higher postnatal blood lead levels and IgE concentrations, after adjusting for age [13]. An additional study of 930 children in Taiwan found blood lead levels to be associated with IgE (β = 0.26 per 1 μg/dL difference, 95% CI 0.009–0.50); after stratifying by sex, adverse associations remained among boys [14]. Furthermore, IgE was examined as a mediator and was estimated that 38% of the total effect of blood lead on asthma was mediated through IgE [14]. The present study adjusted for child sex, child birthweight, maternal race, maternal parity, maternal education, number of cigarettes smoked in the home per day, average adjusted household income, day care attendance, and any breastfeeding by 6 months of age. Comparatively, the results of two of the previous studies may be confounded by many child and maternal characteristics which may explain their positive findings [13, 33].

Limitations of this study include the assessment of asthma via caregiver report of a physician’s diagnosis. Some misclassification may have been introduced if parents or guardians did not recall whether their child had been diagnosed. A study comparing parent-reported asthma diagnoses with health claims (*n* = 2782) observed a moderate Kappa of 0.60 agreement, and a high specificity of correctly identifying a physician asthma diagnosis of 95.9% [37].. These findings suggest that parental report is good, but not excellent, and any resulting non-differential outcome misclassification would have attenuated our results towards the null. Since our study enrolled participants at age 6 months, we were not able to determine whether prenatal blood lead levels were associated with asthma diagnosis. Additionally, the relatively small sample size contributed to wide confidence limits and increased the likelihood of type II errors. Too, the present study could not examine the potential mechanistic link between blood lead levels via measured IgE concentrations. Finally, this study was conducted in an urban sample of children residing in Rochester, NY, born between 1994 and 1995. To the extent that this retrospective sample was exposure to fewer or different environmental chemicals that influence asthma risk than a present-day study, results from the Rochester cohort may be less generalizable.

Our study also had several strengths. Repeated blood lead measurements between the ages of 6–72 months permitted the identification of potential critical periods of exposure, as well as the ability to test average (cumulative) and peak blood lead concentrations as determinants of asthma risk. In addition, the prospective nature of the cohort provided data on important early life confounders such as maternal parity and child birthweight, which might not be readily available in a cross-sectional design. Furthermore, we collected sociodemographic data at multiple time points which permitted construction of variables—such as average adjusted household income—that considered multiple time periods and sources of income, which served to reduce residual confounding, which could otherwise distort the lead-asthma association.

In conclusion, we did not observe an association between child blood lead levels and physician diagnosed asthma by age 72 months in this cohort. Although prenatal lead exposure has been linked with childhood IgE levels, the direct effect of prenatal lead exposure on asthma is not known.

## Data Availability

The datasets used and analyzed during the current study are available on reasonable request to the corresponding author.
